# Comparison of procedural characteristics of percutaneous annuloplasty and edge-to-edge repair for the treatment of severe tricuspid regurgitation

**DOI:** 10.3389/fcvm.2023.1232327

**Published:** 2023-09-08

**Authors:** Isabel Mattig, Fabian Barbieri, Mario Kasner, Elena Romero Dorta, Anna Lisa Heinrich-Schüler, Miry Zhu, Karl Stangl, Ulf Landmesser, Markus Reinthaler, Henryk Dreger

**Affiliations:** ^1^ Department of Cardiology, Angiology and Intensive Care Medicine, Deutsches Herzzentrum der Charité, Campus Charité Mitte, Berlin, Germany; ^2^ Charité – Universitätsmedizin Berlin, Corporate Member of Freie Universität Berlin and Humboldt-Universität zu Berlin, Berlin, Germany; ^3^ DZHK (German Centre for Cardiovascular Research), Berlin, Germany; ^4^ Berlin Institute of Health at Charité – Universitätsmedizin Berlin, BIH Biomedical Innovation Academy, Berlin, Germany; ^5^ Department of Cardiology, Angiology and Intensive Care Medicine, Deutsches Herzzentrum der Charité, Campus Benjamin Franklin, Berlin, Germany; ^6^ Institute of Active Polymers and Berlin-Brandenburg Center for Regenerative Therapies, Helmholtz-Zentrum Hereon, Teltow, Germany; ^7^ Department of Cardiology, Angiology and Intensive Care Medicine, Deutsches Herzzentrum der Charité, Campus Virchow Klinikum, Berlin, Germany

**Keywords:** tricuspid regurgitation, percutaneous annuloplasty, transcatheter edge-to-edge repair, procedural characteristics, transcatheter valve intervention

## Abstract

**Background:**

In recent years, new interventional therapies for tricuspid regurgitation (TR) demonstrated their effectiveness in reducing TR severity and improving symptoms. Currently, tricuspid transcatheter edge-to-edge repair (T-TEER) and percutaneous annuloplasty are the most widely used techniques in Europe. In this retrospective study, we compared procedural characteristics and learning curves of both TR devices in a real-world cohort.

**Material and methods:**

Eligible patients with severe to torrential TR underwent either percutaneous annuloplasty or T-TEER as recommended by the local heart team. Patients with combined mitral and tricuspid interventions were excluded from the analysis. The study focused on procedural characteristics, TR reduction and learning curves.

**Results:**

A total of 122 patients underwent either percutaneous annuloplasty (*n *= 64) or T-TEER (*n *= 58) with a technical and device success rate of 98% and 97%, respectively. Reasons for technical failure included right coronary artery (RCA) dissection prior to percutaneous annuloplasty, and two single leaflet device attachments (SLDA) during T-TEER implantation. The mean improvement of TR severity was 2.4 ± 0.8 degrees after T-TEER and 2.5 ± 0.8 after percutaneous annuloplasty. T-TEER procedures were shorter in terms of both procedure time and radiation exposure, while percutaneous annuloplasty, although taking longer, showed a significant reduction in procedure time over the course of the analysed period.

**Conclusion:**

In summary, both interventional therapies reduce TR severity by approximately two degrees when used in the appropriate anatomy. The learning curve for annuloplasty group showed a significant decrease of procedure times.

## Introduction

Significant tricuspid regurgitation (TR) is a prevalent valvular heart disease that is observed in 4% of patients over 75 years of age (1). TR is mostly secondary due to left heart pathologies, atrial fibrillation, or pulmonary hypertension (1). Even mild to moderate TR leads to a significant increase in morbidity and mortality (2). Recommended surgical repair or replacement in severe symptomatic TR is associated with a high intra-hospital mortality rate of 8%–10% and is rarely performed (3, 4). In recent years, new interventional therapies have been developed to address this clinical need (5). These therapies comprise edge-to-edge repair, annuloplasty devices as well as orthotopic and heterotopic valve implantations (6, 7). Transcatheter edge-to-edge repair (T-TEER) and percutaneous annuloplasty are currently the only approved and consequently most commonly used interventional therapies for tricuspid regurgitation in Europe (5–10). The European Society of Cardiology (ESC) and European Association for Cardiothoracic Surgery (EACTS) suggest interventional TR therapy in inoperable patients (3).

The T-TEER approach primarily diminishes TR by clipping the leaflets, while tricuspid annuloplasty reduces the annular circumference. Consequently, both devices are implanted in varying TR morphologies (5, 7). T-TEER is favored in patients with an anteroseptal jet and a small to moderate gap between the two target leaflets (≤7 mm), preferably in a trileaflet morphology (5, 7). In contrast, best results after annuloplasty are expected in patients with predominantly annular dilatation, none or mild tethering, a central jet and a suitable landing zone to insert the anchors, such as a sufficient distance to the right coronary artery (RCA) (5, 7). Both techniques can be used in case of cardiac implantable electronic devices (CIEDs) (7). However, a superior post-interventional outcome is expected without any right ventricular lead (7). Precondition for a successful procedure is a favorable transesophageal echocardiography (TOE) window or the availability of intracardiac ultrasound (5, 7).

A significant number of patients present with anatomies amenable by either T-TEER or annuloplasty. Therefore, other considerations such as procedure time and potential complications can play a role in the decision-making process for the appropriate device. In addition, as interventional TR repair is still a novel therapy, starting centers might be interested in expected learning curves for both approaches. Thus, the aim of present study was to provide a comparison of procedural characteristics and learning curves of the most widely used TR devices, i.e., percutaneous annuloplasty and T-TEER, in a real-world cohort.

## Materials and methods

### Study design

The retrospective multi-centre study compared percutaneous annuloplasty and T-TEER of the tricuspid valve in a real-world cohort. Patients with an age ≥18 years, high surgical risk and symptomatic severe to torrential TR of different pathologies and despite optimal medical therapy underwent either Cardioband® implantation (Edwards Lifesciences, Irvine, CA, USA) or T-TEER (TriClip®, Abbott, Chicago, Illinois, USA, or PASCAL®, Edwards Lifesciences, Irvine, California, USA) from 2019 to 2022. Patients with combined procedures of the tricuspid and mitral valve were excluded from the analysis. In case of two sequential TR procedures, only the first intervention was evaluated. The study was approved by the institutional ethics committee of the Charité – Universitätsmedizin Berlin, Germany (EA1/005/23).

Interventional therapy of TR was recommended by local heart team consisting of interventional cardiologists, cardiovascular surgeons, anesthetists, heart failure specialists, and imaging experts. The appropriate device was selected based on individual tricuspid anatomy and function according to current recommendations (5, 7). Patients with a systolic pulmonary artery pressure ≥75 mmHg measured by right heart catheterization did not undergo an interventional therapy. Evaluation of tricuspid regurgitation and its interventional therapy comprised an echocardiographic assessment, right and left heart catheterization as well as computed tomography in line with the guidelines of the ESC and EACTS (3). Echocardiography was performed according to the standards of the American Society of Echocardiography (ASE) and the European Association of Cardiovascular Imaging (EACVI) using a GE healthcare Vivid E9 or E95 with a M5S(c) 1.5–4.5 MHz or 4Vc-D 3D/4D Phased Array 1.4–5.2 MHz transducer (GE Vingmed, Horton, Norway) (11, 12). TR was quantified by assessment of the effective regurgitant orifice area (EROA) and regurgitant volume (RegVol) using the proximal isovelocity surface area (PISA) method, vena contracta measurements, as well as hepatic vein reflux. TR severity was graded into five categories as proposed by Hahn et al. (13). TOE or intracardiac ultrasound was used for guidance of the interventional therapy. All procedures were performed as described previously under general anesthesia (8, 10, 14).

Outcome parameters were the following procedural characteristics: technical and device success and complications based on the Mitral Valve Academic Research Consortium (MVARC) criteria (15) as well as TR reduction and learning curves, i.e., changes of outcome and procedural characteristics over time. Technical and device success were combined and defined as successful implantation of the device and retrieval of the delivery system without conversion to open heart surgery or other interventional therapy as well as TR reduction of at least one grade at the time of leaving the operating room (adapted from MVARC criteria). TR grade was quantified at the end of the procedure and at discharge.

### Statistical analysis

SPSS Statistics version 28 for Windows (IBM Corporation, New York, NY, USA) was used for data analysis. Categorical and ordinal variables were listed in percentages and were analysed by chi-squared test or Mann-Whitney *U*-test, respectively. Continuous variables were presented as median with 25th and 75th percentile or mean with standard deviation depending on their skewness and uniform per variable for a better inter- and intragroup comparison. Continuous variables were assessed by *t*-test (normally distributed parameters) and Wilcoxon-test or Mann-Whitney *U*-test (not normally distributed parameters). The impact of TR reduction on right ventricular function and learning curves were evaluated by linear regression analysis. TR improvement (difference between TR grade after intervention and at baseline) was assumed to be a continuous variable. A *p*-value of <0.05 was considered statistically significant.

## Results

**Figure 1:**
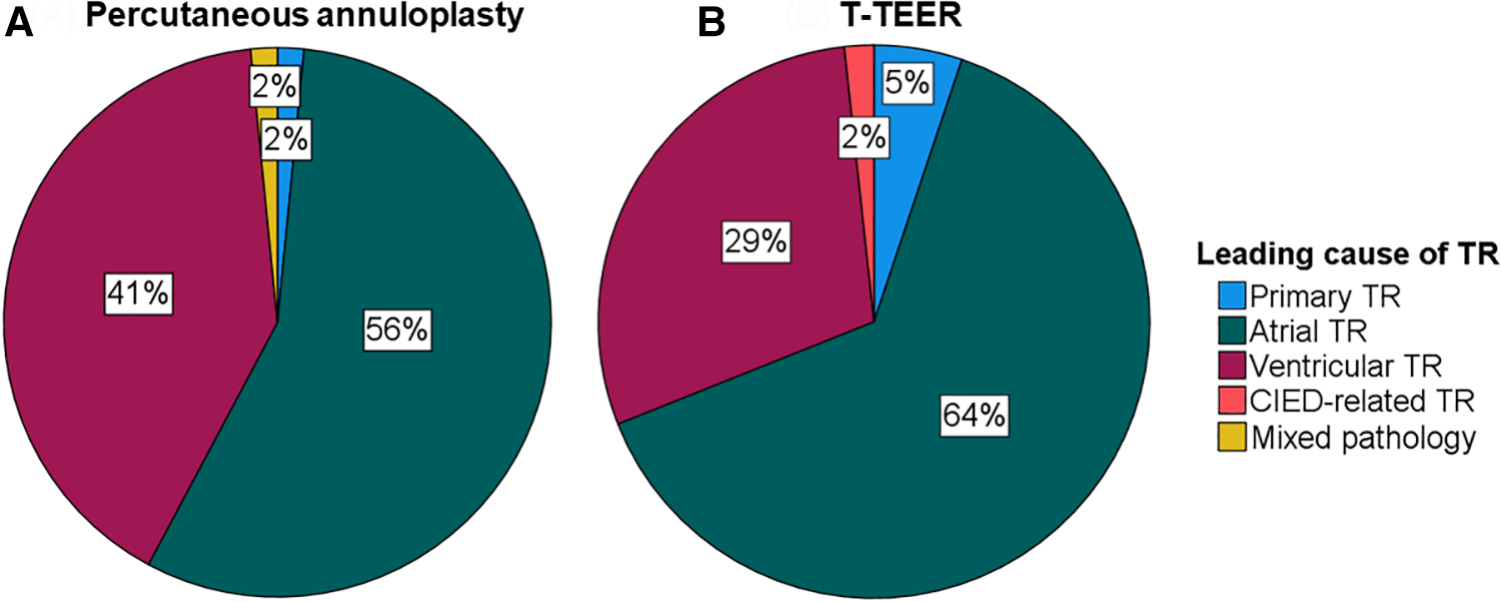
Leading cause of TR of patients undergoing percutaneous annuloplasty (**A**) or T-TEER (**B**). CIED, cardiac implantable electronic device; T-TEER, transcatheter edge-to-edge repair; TR, tricuspid regurgitation.

A total of 122 patients with severe to torrential TR underwent either percutaneous annuloplasty or T-TEER in two centres between 2019 and 2022. Baseline characteristics of the study cohort are listed in Table 1. Patients undergoing percutaneous annuloplasty or T-TEER had a median age of 81 and 82 years, respectively. Overall, 80% of patients reported a New York Heart Association Class (NYHA class) of III or IV, and 31% had a history of CIED implantation including one patient with a leadless pacemaker in the annuloplasty group. In detail, 25 patients (43%) in the T-TEER group and 12 patients (19%) in the annuloplasty group had a right ventricular lead crossing the tricuspid valve at the time of the intervention (*p* = 0.003). There was no significant difference in leading TR pathology between annuloplasty or T-TEER group (*p *= 0.329, Figure 1). Echocardiographic characteristics and pulmonary artery pressures did not differ significantly between the two groups except for tenting height at baseline (Tables 1–3). While the mean left ventricular function was mildly reduced in the T-TEER group, the mean right ventricular function was within a normal range in both cohorts. The mean right ventricular diameter and atrial area were dilated in both groups and patients who underwent annuloplasty had more often a torrential TR at baseline. One patient each from the annuloplasty and T-TEER group received a second intervention due to TR deterioration after 1 year and after 4 months, respectively. Only the first procedures were included in our analysis, as stated before.

**Table 1:** Baseline characteristics.

	Annuloplasty (*n *= 64)	T-TEER (*n *= 58)	*p*-value
Female, *n* (%)	41 (64)	33 (57)	0.408
Age, years (IQR)	81 (77–84)	82 (79–84)	0.386
BMI, kg/m^2^ (IQR)	25 (22–28)	25 (22–28)	0.982
EUROScore II (IQR)	4.3 (3.0–7.5)	5.2 (3.6–9.1)	0.111
NYHA class			0.700
I, *n* (%)	0 (0)	0 (0)	
II, *n* (%)	13 (20)	11 (19)	
III, *n* (%)	47 (75)	42 (72)	
IV, *n* (%)	4 (6)	5 (9)	
Coronary artery disease, *n* (%)	36 (56)	32 (55)	0.905
Percutaneous coronary intervention, *n* (%)	20 (31)	22 (38)	0.438
Coronary artery bypass graft, *n* (%)	2 (3)	6 (10)	0.108
Cardiac implantable electronic device, *n* (%)	13 (20)	25 (43)	0.007
Atrial fibrillation or flutter, *n* (%)	62 (97)	53 (91)	0.192
Arterial hypertension, *n* (%)	55 (86)	47 (81)	0.465
Diabetes mellitus, *n* (%)	17 (27)	16 (28)	0.847
Stroke, *n* (%)	11 (17)	9 (16)	0.803
Peripheral artery disease, *n* (%)	5 (8)	12 (21)	0.040
Chronic obstructive pulmonary disease, *n* (%)	10 (16)	12 (21)	0.467
Asthma, *n* (%)	2 (3)	3 (5)	0.569
Long-term oxygen therapy, *n* (%)	1 (2)	0 (0)	0.339
Dialysis, *n* (%)	2 (3)	0 (0)	0.175
History of surgical or percutaneous valve therapy, *n* (%)	21 (33)	26 (45)	0.173
Serum creatinine, mg/dl (IQR)	1.3 (1.0–1.5), *n *= 62	1.3 (1.0–1.7)	0.576
NT-proBNP, ng/L (IQR)	2,746 (1,757–4,395), *n *= 59	2,252 (1,275–4,135), *n *= 57	0.243
Alanine aminotransferase, U/L (IQR)	19 (17–25)	21 (16–27), *n *= 57	0.730
Aspartate aminotransferase, U/L (IQR)	31 (26–39)	32 (27–38), *n *= 57	0.952
Gamma glutamyl transferase, U/L (IQR)	91 (53–162), *n *= 60	67 (45–124), *n *= 53	0.157
Bilirubin, mg/dl (IQR)	0.6 (0.5–1.0), *n *= 36	1.0 (0.5–1.6), *n *= 46	0.041
Systolic PAP, mmHg ± SD	42 ± 14, *n *= 34	40 ± 14, *n *= 37	0.482
Heart failure therapy			
Diuretics, *n* (%)	60 (94)	58 (100)	0.053
Beta-blocker, *n* (%)	55 (86)	52 (90)	0.532
ACE inhibitor or sacubitril/valsartan, *n* (%)	50 (78)	41 (71)	0.346
Mineralocorticoid receptor antagonist, *n* (%)	35 (55)	31 (53)	0.891
SGLT2 inhibitor, *n* (%)	15 (23)	20 (35)	0.178

Continuous variables are shown as median and interquartile ranges (IQR, not normally distributed), categorical variables are given as absolute number with percentages. T-TEER, transcatheter edge-to-edge repair; BMI, body mass index; EUROScore II; European System for Cardiac Operative Risk Evaluation II, NYHA class, New York Heart Association Class; NT-proBNP, N-terminal pro brain natriuretic peptide; PAP, pulmonary artery pressure; ACE, angiotensin-converting enzyme; SGLT2, sodium-glucose transport protein 2.

**Table 2:** Echocardiographic characteristics at baseline.

	Annuloplasty (*n *= 64)	T-TEER (*n *= 58)	*p*-value
LVEF, % ± SD	55 ± 10	50 ± 10	0.132
RVD basal, mm ± SD	47 ± 8	49 ± 10	0.332
RVD mid, mm ± SD	36 ± 9, *n *= 63	38 ± 10, *n *= 56	0.148
RA area, cm^2^ ± SD	35 ± 10	36 ± 10	0.601
TAPSE, mm ± SD	18 ± 5, *n *= 63	17 ± 4, *n *= 56	0.361
RVFAC, % ± SD	40 ± 9	41 ± 11, *n *= 54	0.487
EROA, cm^2^ (IQR)	0.7 (0.5–1.3), *n *= 62	0.6 (0.5–0.9), *n *= 54	0.062
Vena contracta, mm ± SD	15 ± 5	13 ± 4	0.057
Tenting height, mm ± SD	6 ± 5	4 ± 4	<0.001
Coaptation gap ATL-STL, mm (IQR)	4 ± 3, *n *= 42	5 ± 2, *n *= 52	0.222
Coaptation gap PTL-STL, mm (IQR)	4 ± 2, *n *= 42	5 ± 2, *n *= 53	0.150
Mean annular diameter^a^, mm	43 ± 5	41 ± 5	0.118

Continuous variables are shown as mean ± standard deviation (SD, normally distributed) or median with interquartile ranges (IQR, not normally distributed). T-TEER, transcatheter edge-to-edge repair; LVEF, left ventricular ejection fraction; RVD, right ventricular diameter; RA area, right atrium area; TAPSE, tricuspid annular plane systolic excursion; RVFAC, right ventricular fractional area change; EROA, effective regurgitant orifice area; ATL, anterior tricuspid leaflet; STL, septal tricuspid leaflet; PTL, posterior tricuspid leaflet.

^a^ The average of the “end diastolic” septolateral and anterior-posterior tricuspid annulus diameters, at transoesophageal echocardiography.

**Table 3:** Echocardiographic characteristics of patients with complete measurements at baseline and discharge.

	Annuloplasty (*n *= 60)		*p*-value for comparison baseline vs. discharge (annuloplasty)	T-TEER (*n *= 57)		*p*-value for comparison baseline vs. discharge (T-TEER)	*p*-value for comparison of annuloplasty vs. T-TEER at discharge
	Baseline	Discharge		Baseline	Discharge		
LVEF, % (IQR)	55 (50–59)	55 (50–60)	0.361	51 (45–55), *n *= 56	55 (52–59), *n *= 56	<0.001	0.827
RVD basal, mm ± SD	47 ± 9, *n *= 53	45 ± 9, *n *= 53	0.159	49 ± 10, *n *= 56	46 ± 10, *n *= 56	0.008	0.838
RVD mid, mm ± SD	37 ± 9, *n *= 50	37 ± 9, *n *= 50	0.659	38 ± 10, *n *= 52	36 ± 10, *n *= 52	0.107	0.383
RA area, cm^2^ (IQR)	33 (29–40)	29 (25–35)	<0.001	34 (28–43)	33 (27–40)	0.007	0.116
TAPSE, mm ± SD	19 ± 5, *n *= 58	15 ± 4, *n *= 58	<0.001	17 ± 4, *n *= 51	17 ± 4, *n *= 51	0.765	0.002
RVFAC, % ± SD	39 ± 7, *n *= 43	35 ± 9, *n *= 43	0.004	41 ± 11, *n *= 43	36 ± 9, *n *= 43	0.003	0.699

Continuous variables are shown as mean ± standard deviation (SD, normally distributed) or median with interquartile ranges (IQR, not normally distributed). T-TEER, transcatheter edge-to-edge repair; LVEF, left ventricular ejection fraction; RVD, right ventricular diameter; RA area, right atrium area; TAPSE, tricuspid annular plane systolic excursion; RVFAC, right ventricular fractional area change.

### Procedural characteristics and complications

Technical and device success was achieved in 97% of patients in the T-TEER group and 98% of patients in the annuloplasty group (*p *= 0.502). Reasons for device failure were two single leaflet device attachments (SLDA) after T-TEER. Two additional SLDA occurred during the intra-hospital stay. One patient with planned Cardioband® implantation developed cardiac arrest due to iatrogenic coronary dissection after engagement of the right coronary artery, necessitating cardiopulmonary resuscitation. Therefore, the Cardioband® implantation was aborted. We did not include this case in the further analysis of procedural characteristic and durable repair. None of the patients died during the procedure. One patient of the annuloplasty group suffered from a major vascular access complication, a dissection of the left iliac artery due to pronounced kinking, treated by stent implantation. The implantation of the Cardioband® was performed during a second procedure without further complications.

**Figure 2:**
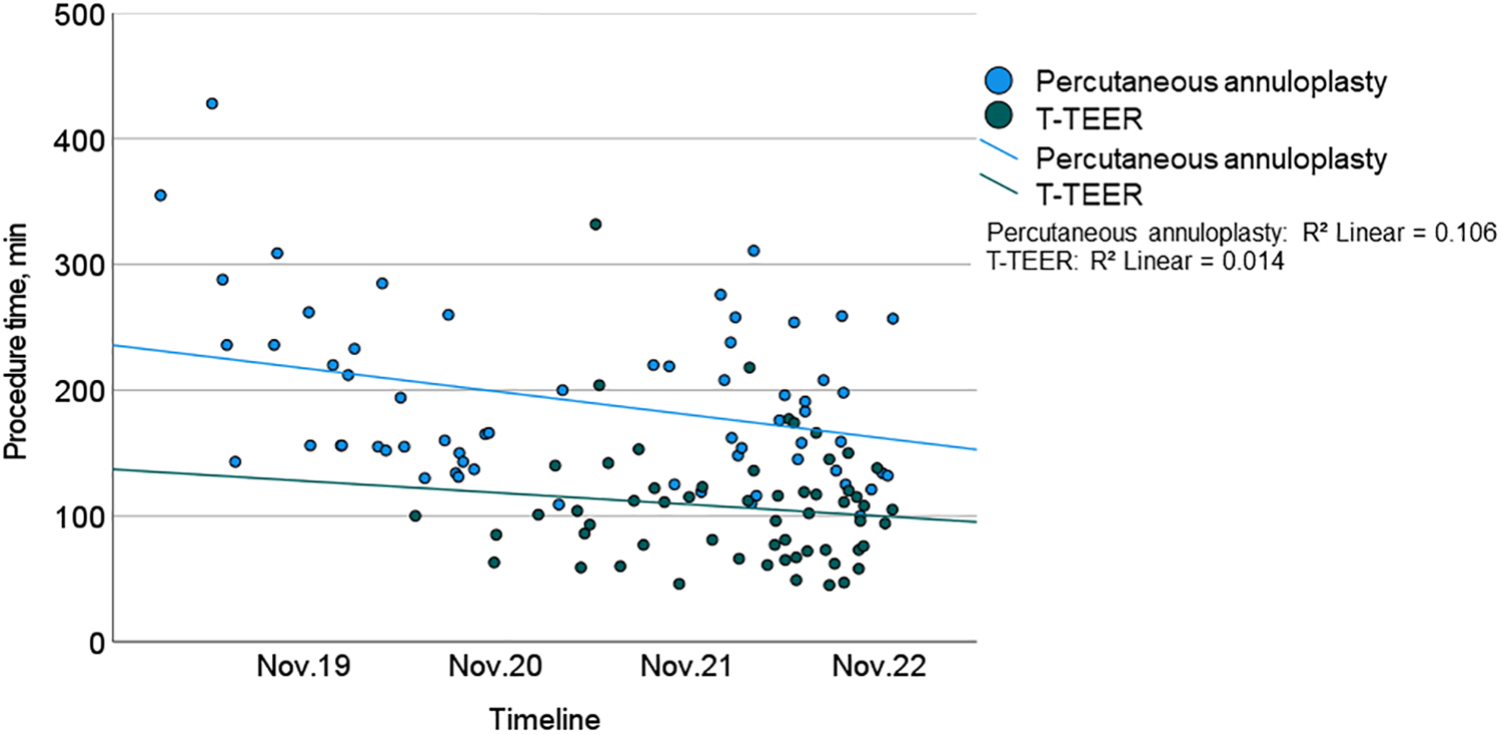
Procedure time of T-TEER (green) and percutaneous annuloplasty (blue) including trend lines during the study from 2019 to 2022. T-TEER, transcatheter edge-to-edge repair.

**Figure 3:**
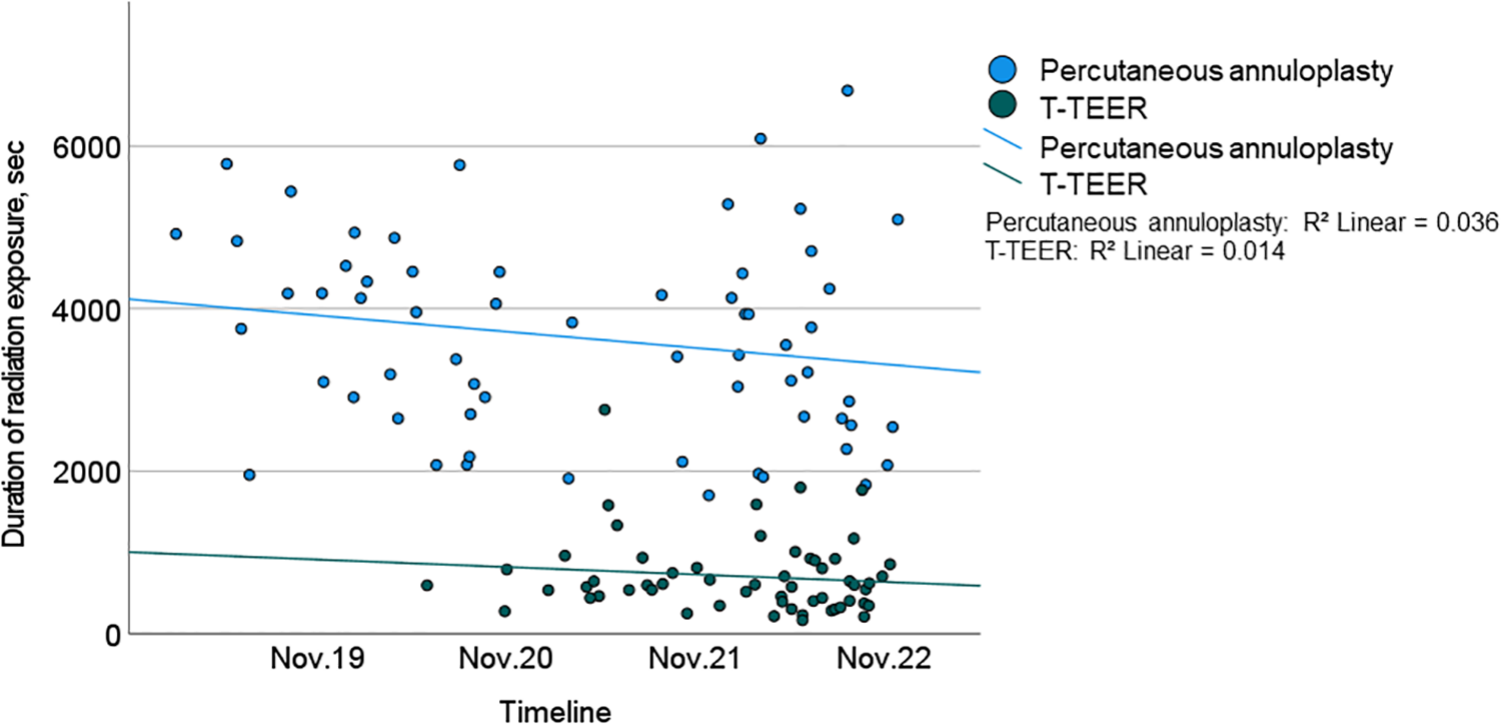
Duration of radiation exposure of T-TEER (green) and percutaneous annuloplasty (blue) including trend lines over the course of the study (2019–2022). T-TEER, transcatheter edge-to-edge repair.

Procedure time and duration of radiation exposure were significantly shorter in the T-TEER group compared to the annuloplasty group (T-TEER: 102 [73–122] min and 10 [7–15] min vs. annuloplasty: 165 [143–236] min and 61 [44–74] min, *p *< 0.001; Figures 2, 3). On average, 1.9 ± 0.6 devices per patient were implanted in the T-TEER group, mostly into the anteroseptal, followed by the posteroseptal commissure. Annuloplasty devices comprised the sizes C to F. On average, 121 ± 62 ml of contrast were used for Cardioband® implantation compared to none for T-TEER. Annuloplasty patients presented significantly more often a new or progressive but hemodynamically not relevant pericardial effusion compared to patients after T-TEER. Moreover, four cases of transient bradycardia and three incidents of right coronary artery injuries requiring intervention occurred during percutaneous annuloplasty. Detailed procedural complications are listed in Table 4.

**Table 4:** Procedural complications.

	Annuloplasty (*n *= 63)	T-TEER (*n *= 58)	*p*-value
Injury of the right coronary artery requiring intervention, *n* (%)	3 (5)	0 (0)	0.092
Transient bradycardia, *n* (%)	4 (6)	0 (0)	0.051
Single leaflet device attachments, *n* (%)	0 (0)	4 (7)	0.034
Pericardial effusion, *n* (%)	17 (27)	3 (5)	0.001
Access complication	1 (2)	0 (0)	0.335

Categorical variables are given as absolute number with percentages. T-TEER, transcatheter edge-to-edge repair.

### Reduction of tricuspid regurgitation and its impact on right heart morphology and function

**Figure 4:**
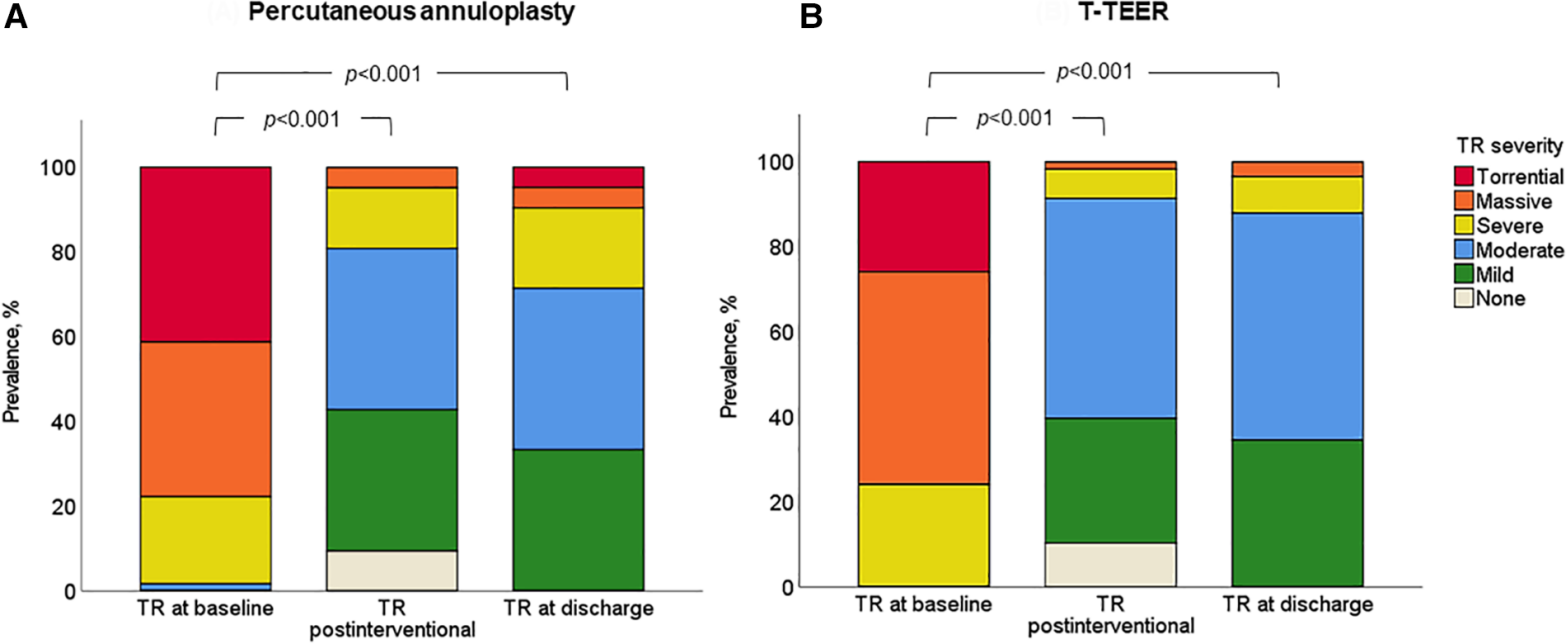
Severity of TR in the annuloplasty (**A**) and T-TEER group (**B**) at baseline, postinterventional and at discharge (*p *< 0.001 for comparison of baseline and postinterventional as well as baseline and discharge in both groups). T-TEER, transcatheter edge-to-edge repair; TR, tricuspid regurgitation.

**Figure 5:**
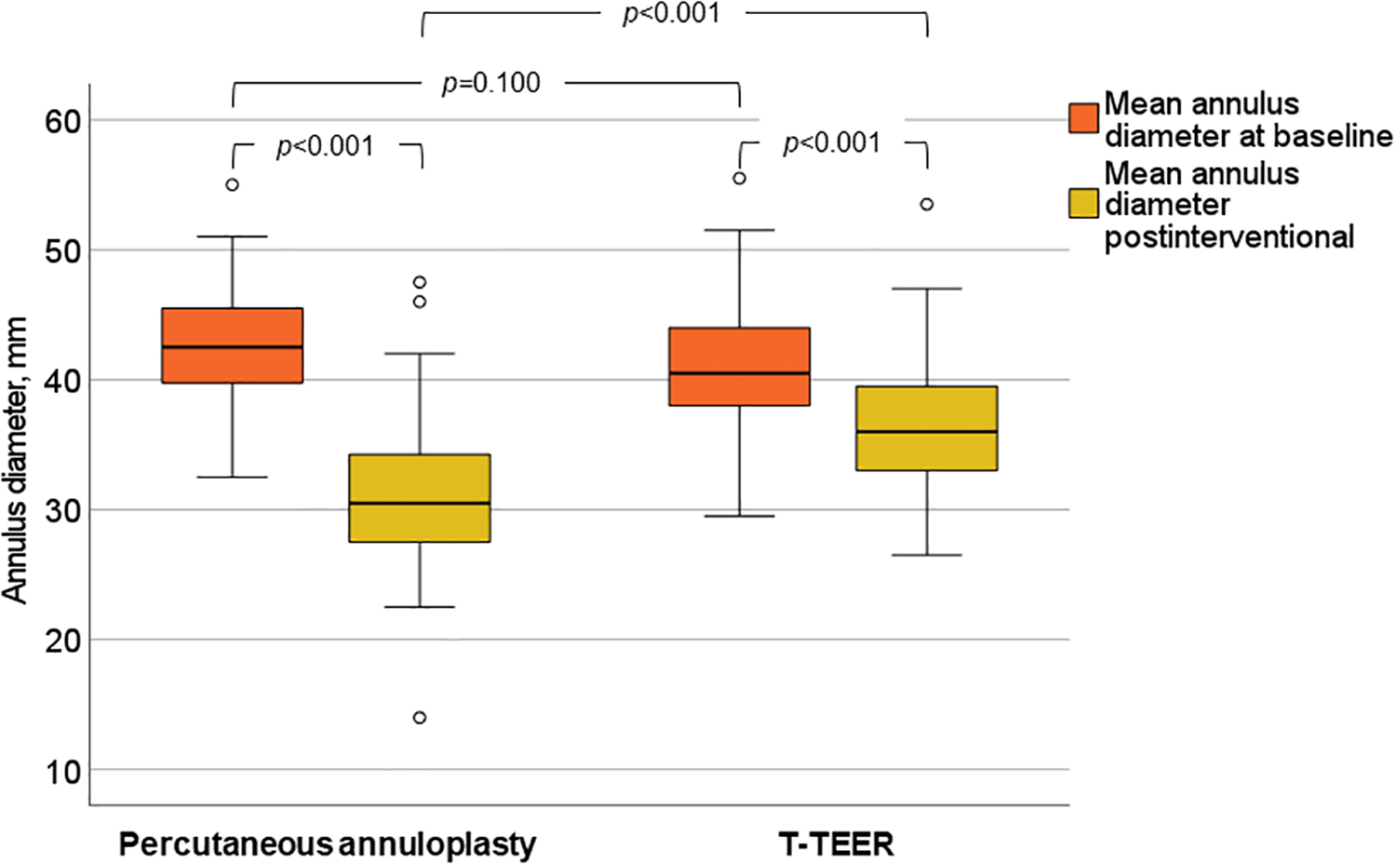
Mean annulus diameter of the tricuspid valve before and after annuloplasty and T-TEER. T-TEER, transcatheter edge-to-edge repair.

TR was reduced at least one grade in 100% of patients, with 91% of T-TEER patients and 80% of annuloplasty patients achieving a TR grade ≤2 post-intervention. The mean reduction was 2.4 ± 0.8 grades after T-TEER and 2.5 ± 0.8 grades after annuloplasty (Figure 4). Moreover, we observed a significant decrease in annulus diameter in both groups after the procedure, which was more pronounced after annuloplasty (Figure 5). Grading of TR severity by transthoracic echocardiography at discharge was consistent with the intraprocedural assessment at the end of the intervention in 69% of patients in the T-TEER group and in 64% of patients in the annuloplasty group (*p* = 0.650 for comparison of both groups).

In both groups, we observed a significant decrease of right ventricular function as assessed by right ventricular fractional area change (RVFAC) and a significant reduction of right atrial area, while tricuspid annular plane systolic excursion (TAPSE) was only reduced after annuloplasty (Table 3). Moreover, we detected a significant impact of TR reduction on RVFAC after T-TEER, but not after annuloplasty (linear regression analysis for T-TEER: *F*-value 7.7, *p *= 0.008, regression coefficient 46.1, *p *< 0.001).

### Learning curve

The procedure time of annuloplasty showed a significant reduction throughout the study period. There was neither a relevant effect of a learning curve on TR reduction in both groups nor on procedure time in the T-TEER group.

## Discussion

The present study compares procedural characteristics and learning curves of percutaneous annuloplasty and T-TEER in a real-world setting. Both interventional therapies reduced TR severity by approximately two degrees. Technical and device success was achieved in 97% of patients in the T-TEER and 98% of patients in the annuloplasty group. Complications during percutaneous annuloplasty comprised intermittent bradycardia and injuries of the right coronary artery. SLDA occurred in 7% of patients after edge-to-edge repair. Overall, however, the number of clinically relevant complications was low.

### TR reduction

**Figure 6:**
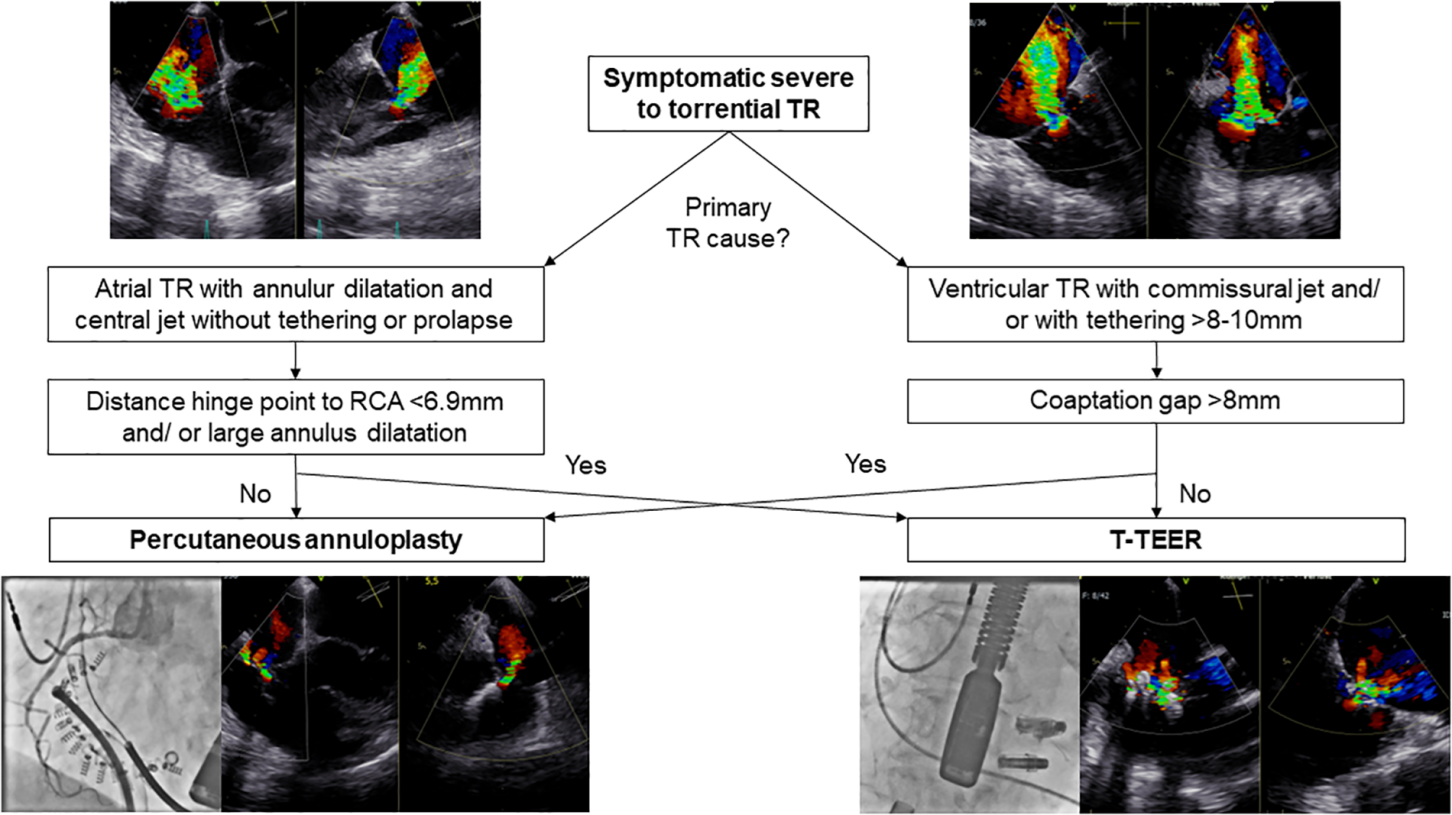
Flow chart for selecting the appropriate device in different TR anatomies. RCA, right coronary artery; T-TEER, transcatheter edge-to-edge repair; TR, tricuspid regurgitation.

TR reduction of at least one grade in 100% of patients in our study cohort is comparable to results of previous T-TEER and annuloplasty trials (8, 14, 16, 17). Device selection was based on tricuspid anatomy and function assessed by echocardiography, computed tomography, and right heart catheterization (5, 7, 8, 16, 17). Figure 6 illustrates our algorithm for device selection, which aligns with similar recommendations published in the literature (5). Although percutaneous annuloplasty is primarily applied in annulus dilatation in atrial TR, TR etiologies did not differ significantly between both groups and annuloplasty as well as T-TEER resulted in a significantly reduced tricuspid annular diameter. Interestingly, the algorithm is not well reflected in the baseline measurements which in fact showed a greater tenting height in the annuloplasty group and a wider coaptation gap in the T-TEER group. The reason for this apparent discrepancy is that a substantial number of cases (*n *= 10) could not be treated with the preferred device—either due to RCA proximity (for annuloplasty) or due to gap size (for T-TEER). This resulted in patients with large gaps (often associated with larger tenting height) being treated by annuloplasty especially in the early phase of the study period. Similarly, patients with suitable valve anatomy for annuloplasty had to undergo T-TEER when RCA proximity made annuloplasty not feasible. With the availability of larger edge-to-edge devices, e.g., TriClip G4 and PASCAL Ace, we observed a reduction of tenting height in the annuloplasty group and an increase of baseline gap size in T-TEER patients. In both groups, patients had right ventricular leads. Lurz et al. already reported comparable efficacy of T-TEER in patients with and without right ventricular leads (7, 18). However, TR improvement was more pronounced in patients with a commissural or central lead compared to a lead in contact to the leaflet body (18). In line with the results by Lurz et al., we also observe no significant difference in TR reduction in patients with or without right ventricular leads in both groups. We observed a decrease in right ventricular function, as measured by TAPSE, after interventional therapy, possibly driven by reduction of regurgitation volume in both groups and the annuloplasty device itself. Therefore, we recommend further follow-up visits including assessment of functional status, medical therapy, and echocardiography. This is necessary to prevent the potential oversight of clinical deterioration associated with an afterload mismatch and the decrease in right ventricular function. At baseline, the majority of our patients were either receiving diuretic treatment or undergoing dialysis in addition to heart failure therapy (Table 1). It is advisable to adjust diuretic therapy depending on venous congestion during the follow-up visits.

### Procedural complications

Intermittent bradycardia occurred only in the annuloplasty group, most likely due to mechanical compression on the conduction system in the region of Koch's triangle (19). While the observed bradycardia was usually self-limiting and of short duration, operators should be aware of this possible complication occurring during placement of the last anchors. Compression or injury of the right coronary artery (RCA) requiring percutaneous coronary intervention after anchor placement (5% of our patients) was mostly caused by severe proximity of the RCA to the tricuspid annulus. In addition, we found new or progressive pericardial effusions in 27% of patients after percutaneous annuloplasty, which were most likely due to pericardial irritation resulting from anchor insertion. The pericardial effusions were usually constant until discharge and had no clinical relevance.

In the T-TEER cohort, four SLDA occurred during the procedure (*n *= 2) and up to discharge (*n *= 2) between the posterior and septal (*n *= 2) as well as the anterior and septal leaflets (*n *= 2). All T-TEER patients with SLDA presented a massive TR with degenerative and short leaflets as well as a larger tricuspid annulus diameter at baseline (45 [44–49] mm) compared to those without SLDA (40 [38–44] mm). However, the tenting height and coaptation gaps were comparable between the two groups, measuring approximately 4 ± 3 mm (*n *= 4) and ranging from 2 to 5 mm (*n *= 3) in the SLDA group, respectively. All patients underwent a re-intervention (T-TEER) resulting in a moderate (*n *= 1), severe (*n *= 2) or massive TR (*n *= 1) at discharge. Based on these findings, we propose that the combination of shortened and degenerative leaflets, along with excessive annular dilation, may represent potential risk factors for the development of SLDAs in T-TEER. SLDA is a well-known complication in tricuspid and mitral edge-to-edge repair (20–22). Known risk factors comprise a big coaptation gap between both target leaflets, a severe tethering resulting in a small leaflet insertion and a high mechanical stress on the target leaflet caused by the devices as well as the leaflet tissue itself (20, 22, 23). Current strategies to treat residual TR include stabilization of the device by implanting another device or, in case of no available landing zone, the implantation of a cava valve (24, 25).

### Learning curve

We observed a significant learning curve only in the annuloplasty group in terms of procedural duration. Specifically, the first studies of Cardioband® implantation showed a mean procedure time of 4.2 h (8), which was significantly longer than the mean procedure time of 2.8 h in our cohort. This suggests that further development of annuloplasty devices, the use of intracardiac ultrasound and increasing experience of interventional cardiologists may enable further reduction in procedure times. However, it is likely that percutaneous annuloplasty will continue to take longer than T-TEER, as it involves significantly more procedural steps. In the T-TEER group, the duration of procedures also tended to get shorter during the study period, but this did not reach statistical significance. There was no relevant change in the number of implanted devices over time. No further improvements in terms of a learning curve were observed during the study period, in particular no improvement in TR reduction in either group.

In summary, the following aspects might be considered when selecting patients for TR procedure beyond anatomical characteristics: The longer procedure time of percutaneous annuloplasty results in longer duration of general anesthesia and mechanical ventilation, which might increase complications such as pneumonia due to microaspiration and prolonged ventilation as well as delirium in older patients. The implantation requires the use of contrast, which should be taken into account in patients with renal failure including patients with cardiorenal syndrome. However, more severe TRs were treated successfully with annuloplasty compared to T-TEER. T-TEER might be challenging in patients with a big coaptation gap leading to an increased risk of SLDA. Nevertheless, percutaneous annuloplasty and T-TEER resulted in a comparable TR reduction in our study cohort. Long-term data for both devices revealed a durable TR improvement, which was associated with symptoms relief and reduction of mortality and heart failure hospitalizations (19, 26–28). However, prospective, randomized trials are needed to better understand pros and cons of both devices and its use in different TR etiologies.

### Limitations

The present study utilized a retrospective design, and patients were not randomized to specific treatment options. Device selection was based on the TR anatomy. As a result, baseline characteristics showed differences in three aspects: presence of CIEDs, bilirubin levels, and peripheral artery disease. These differences reflect real-world data. Therefore, the present study serves as a hypothesis-generating investigation for further randomized-controlled trials.

## Conclusion

To conclude, both T-TEER and percutaneous annuloplasty reduce TR severity by approximately two degrees when used in the appropriate anatomy without clinically significant complications. The learning curve of the annuloplasty group presented a significant decrease of procedure time, which might be a relevant aspect for starting centres.

## Data Availability

The raw data supporting the conclusions of this article will be made available by the authors, without undue reservation.
